# The association between exposure and psychological health in earthquake survivors from the Longmen Shan Fault area: the mediating effect of risk perception

**DOI:** 10.1186/s12889-016-2999-8

**Published:** 2016-05-18

**Authors:** Jiuping Xu, Jiuzhou Dai, Renqiao Rao, Huaidong Xie

**Affiliations:** Institute of Emergency Management and Reconstruction in Post-disaster, Sichuan University, Chengdu, China

**Keywords:** Exposure, Psychological health, Risk perception, Post-earthquake

## Abstract

**Background:**

In this study, exposure refers to survivors who suffered from life-changing situations, such as personal injuries, the deaths or injury of family members, relatives or friends or the loss of or damage to personal or family property, as a result of the earthquake. The mediating effect of risk perception on the exposure and psychological health in survivors from the Longmen Shan Fault area and the moderating effect of social support on the relationship between risk perception and psychological health were both examined.

**Method:**

A cross-sectional survey was conducted in a local Longmen Shan Fault area near the epicenter of the 2008 Wenchuan earthquake. The Diagnostic and Statistical Manual of Mental Disorders, Fourth Edition (DSM-IV), the standard Chinese 12-item Short Form (SF-12v2), and the Social Support Rating Scale (SSRS) were used to interview 2,080 earthquake survivors in the period one-year after the earthquake. Based on the environment and the characteristics of the Longmen Shan Fault area, a risk perception questionnaire was developed to evaluate survivor risk perception. Factor and regression analyses were conducted to determine the hypothetical relations.

**Results:**

The analyses provided effective support for the hypothesized model. Survivor risk perception was classified into direct risk perception and indirect risk perception. Survivor direct risk perception was found to play a partial mediating role in the relationship between exposure and the two domains (Physical component summary (PCS) and the Mental component summary (MCS)) of psychological health. Survivor indirect risk perception was found to have a only partial mediating effect on the association between exposure and MCS. Social support was found to moderate the influence of risk perception on psychological health.

**Conclusion:**

Risk communication should be considered in future post-earthquake psychological assistance programs and social support strategies could also be useful in improving psychological health.

## Background

Many researchers, advocates and clinicians have underscored the important relationship between exposure to environmental disasters and mental health. Focus on these issues has become even more important because of the scale of natural disasters that have affected many areas in the world [1]. It has been found that people exposed to disaster events are far more likely to develop psychiatric disorders such as substance abuse, major depression, Post-traumatic Stress Disorder (PTSD) and psychosomatic illnesses [2]. Earthquakes are one of the most destructive natural disasters as they cause significant casualties and property losses and also have long-term psychological and physiological effects on survivors [3]. Consequently, post-disaster mental health, and especially PTSD [7–11], has become the focus of significant recent research [4–6]. Demographic characteristics, level of exposure, previous psychological problems and social support have been identified as important indicators for post-disaster psychological problems [12–15]. Some research has also found that the perception of risk or threat can be a predictor of poor post-disaster mental health [16–18]. For example, when people are unable to control their present situation, or when the future cannot be predicted, and these situations continue for a long time, they are often unable to overcome their fear or pain, and feel helpless, lose confidence and become pessimistic [67]. Öhman and Mineka (2001) found that excessive or long-term fear, including the fear of imaginary disaster events, caused long–term stress to the mind and body resulting in serious physiological and psychological problems [68]. These findings provided important theoretical contributions to post-disaster mental health research, and also suggested valuable psychological interventions for survivors. However, there are complex relationships within these elements. For example, survivor risk perception has been found to be often affected by socio-economic factors, disaster-exposure characteristics, cultural contexts and personality characteristics, so the structure of perceived risk is not the same in every situation [19–22]. For this reason, it has been speculated that there may be a more complex relationship between risk perception, disaster-exposure and mental health. In the relationship between exposure and mental health, the simple “exposure-mental health” model can be further refined and perfected to detail the generation mechanism for post-disaster psychological health problems, and determine the important role risk perception plays. This work contributes to post-disaster psychological health research, offers new ideas for focused psychological intervention, and provides targeted solutions for survivors.

Past research has found a significant negative correlation between earthquake exposure and psychological health (e.g. PTSD) [24, 26–29]. Xu and Song in a study into PTSD in Wenchuan earthquake survivors found that a higher than average exposure predicted a higher prevalence for PTSD [11]. Carson et al. also demonstrated that exposure to injuries or death could lead to a higher degree of PTSD [10]. Because of this significant relationship, appropriate intervention approaches have been designed based on the vulnerability of the exposed groups, such as low-income mothers, children and hospital employees, as well as perceived social support and perceived benefit [4, 12, 13, 15].

Survivors are likely to elevate their judgment of the probability of a disaster and the severity of its consequences because they have already experienced such a disaster [19, 30]. Earthquake exposure affects survivor risk perception, which could further led to emotional and psychological reactions, such as fear, helplessness, sadness, anxiety, depression, and loss of confidence [31–35]. If people at risk remain in high pressure environments, the duration of their anxiety and tension lengthens and can result in mental health problems, especially after catastrophic natural disasters. Further, earthquake risk perception causes people to feel great panic and fear [16]. There are also complex relations between exposure, risk perception and mental health and factors such as demographics, as certain disaster characteristics and levels of disaster-exposure can also be predictors for psychological health problems, which in turn, influence disaster risk perception. With all this in mind, it could be hypothesized that risk perception plays a mediating role between level of exposure and mental health. Once this relationship is proved to exist, then, to perfect the existing post-disaster psychological health theories, some new measures for psychological intervention and disaster management could be developed.

The relationship between social support and mental health has been a main focus in psychology as social support has been found to have an effect on post-earthquake psychological health, with low social support having been found to be a strong predictor of poor mental health [25, 37–39]. Brewin recognized three PTSD risk factors; however, the lack of social support was found to have a somewhat stronger effect than other factors [9]. In our previous Wenchuan earthquake research, low social support was found to be the strongest predictor for PTSD [11]. Conversely, good social support can be a pathway to the relief of mental stress. Zhao et al. in their research emphasized the importance of developing suitable social support strategies to improve the quality of life in earthquake survivors with PTSD symptoms [40]. Maltais et al. examined the mediating or moderating influences of social support in 177 disaster victims after a serious flood [41], and found that risk perception and social support had a significant negative correlation [42]. Therefore, social support may play a moderating role in the link between risk perception and mental health. However, when risk perception is confirmed as a mediator of the relationship between level of exposure and mental health, the moderating effect of social support can also be clarified. Further, through a combination of social support and risk communication, it is possible to propose interventions to mitigate the negative effects of earthquake exposure on the mental health of survivors.

This study mainly investigates the degree of exposure (assessed using a 2-point scale (yes = 1 and no = 0) to measure whether participants experienced some disaster events) of survivors from the 2008 Wenchuan earthquake (which occurred in the Longmen Shan Fault area), their mental health status one year after this event, and their risk perception and level of social support.

The Longmen Shan Fault is on the eastern edge of the Qinghai-Tibet Plateau and the western edge of the Sichuan Basin so does not cover a wide area but it is adjacent to the densely populated Sichuan basin. An earthquake struck Wenchuan in this area on May 12, 2008, measuring 8.0 on the Richter scale. According to the Ministry of Civil Affairs, more than 70,000 were confirmed dead and more than 400,000 were injured, with 18,467 listed as missing and about 6.5 million people left homeless. To develop effective interventions in this area, insight into the correlates of psychological health and the possible mediating factors is necessary. A large-scale survey (*N* = 2,080) was conducted in this area 1 year after this event. Through this investigation we sought to estimate the mediating effect risk perception had on the relationship between exposure level and mental health and the moderating role social support had on risk perception and mental health.

## Method

### Participants

A cross-sectional survey was conducted from May to September 2009 on participants from severely affected counties in two provinces in the earthquake-stricken area. Eighteen counties were selected in Sichuan Province: Dujiangyan, Pengzhou, Chongzhou, Shifang, Mianzhu, Jiangyou, Anxian, Pingwu, Beichuan, Jiange, Qingchuan, Hanyuan, Wenchuan, Lixian, Maoxian, Songpan, Heishui, and Xiaojin. Lueyang was selected in Shaanxi province.

The survey team was divided into 19 small groups, with each group consisting of 2 graduate students and a staff member from the local Branch of the Association for Science and Technology. Specialists from our project and professional doctors from West China Hospital conducted a 5-day training program for the whole team, which included lectures describing the study protocol and instruments, role-play interviews and mutual discussion. A pilot test was carried out with a group of randomly selected survivors before the formal investigation. Minor modifications and adjustments to the language and expression were made from the pilot test feedback. The final version of the questionnaire was used in the formal investigation, for which the sample size was calculated from the following formula for cross sectional surveys [65, 66]:

$$ \mathrm{size}=\frac{Z_{1-\alpha /2}^2P\left(1-P\right)}{d^2}=\frac{1.96\times 0.5\left(1-0.5\right)}{0.05^2}=384 $$

However, considering the objectives of the project, we sought to obtain a much larger sample from across all 19 counties, in which the target population was more than 2 million. The trained groups were assigned to the counties based on a pre-arranged schedule which specified which counties each group was to investigate. Respondents from houses or temporary accommodation or tents were randomly selected according to their birth date. Those who had experienced the earthquake and were aged from 18 to 65 years were interviewed, but people with mental disabilities or who suffered from major psychoses (e.g. schizophrenia, major depressive disorder, organic mental disorders) were excluded. Participants were given information orally about the study purpose before each interview. Some survivors declined to be involved as they were wary of such earthquake surveys and wished to avoid talking about the event. If this was the case, the next closest respondent was invited. Very few respondents had low education levels or had literacy problems. However, those who had problems in noting down answers were assisted by the team members who accompanied them through the survey–completion process. To ensure privacy, interviewers and participants were encouraged to complete the questionnaires in private. A total of 2300 individuals were involved in this survey, with 2080 questionnaires being completed, a response rate of 90.4 %. This study was approved by the Ethics Committee of Sichuan University, and written informed consent was given by all respondents.

### Measures

The self-report questionnaire had four data sets; demographic characteristics, level of exposure, risk perception and mental health, and social support.

#### Demographic characteristics and exposure

Demographic characteristics such as age, gender, level of education, monthly income, and housing status were collected. Age was divided into four groups: 18–30 (coded as 1), 31–40 (coded as 2), 41–50 (coded as 3), and older than 51 (coded as 4). Gender was coded as 1 (male) and 2 (female). The level of education was coded as 1 (no degree), 2 (Bachelor) and 3 (Graduate). There were 4 monthly income levels; 1 = 1,000 Yuan, 2 = from 1,000 to 2,000 Yuan, 3 = from 2,000 to 3,000 Yuan, and 4 = more than 3,000 Yuan. Housing status was coded as: 1 = original house, 2 = public dormitory, 3 = rented house, and 4 = temporary settlements.

Some earthquake situations were used to measure the participants’ exposure levels; the choices were personal injury, deaths or injury of family members, relatives or friends, loss of or damage to personal or family property, being a witness to other people being seriously injured or killed, changing jobs after the earthquake, and relocation to temporary shelters. Exposure degrees were coded as; low exposure (0–1 event), moderate exposure (2–3 events), and high exposure (more than 4 events) groups.

#### Risk perception

A questionnaire was designed to evaluate how survivors in the Longmen Shan Fault area perceived risk after the Wenchuan earthquake. The approach used to investigate ecological risk perception was built on a psychometric paradigm that has been extensively used to characterize human health risk perceptions [19, 45]. In this study, research was conducted into survivor risk perception from Temporal (post-earthquake), Spatial (affected area), social, economic and ecological aspects, which were identified from discussions with experts from disaster, psychological and risk research fields. After a deep investigation of 50 survivors from post-seismic disaster areas, seven risk items were included in the 39 questions, of which questions 1–4 examined risk perceptions of earthquake recurrence, questions 5–11 examined risk perceptions of a secondary disaster occurrence, questions 12–16 examined the risk of post-seismic physical and mental health, questions 17–22 examined the risk of post-seismic unreasonable compensation, questions 23–28 examined the risk of falling into poverty, questions 29–34 examined the risk of ecological environmental deterioration, and questions 35–39 examined the risk of national culture loss.

Based on a previous risk perception study [19], four common risk attributes were examined to assess the risk perceptions; disaster controllability, seriousness of the consequences, occurrence likelihood and familiarity. The questionnaire was first used in the pilot test, and the reliability and validity were confirmed through effectiveness and credibility tests.

#### Mental health

Mental health was estimated using the SF-12-v2 (Cronbach’s alpha = 0.85), which is a suitable measure for large group studies (greater than *n* = 500). Further information was gather using the Short Form-36 Health Survey Summary Scores (Physical Component Summary (PCS) + Mental Component Summary (MCS)), which has been proved valid for Chinese populations in several previous studies [46, 47], has good reliability and validity, correlates well with clinical assessments of physical and mental health [48–50], and has been used in numerous studies worldwide, including China.

A better health state had a higher item score. A T-scores algorithm was designed to convert the psychological items to standard scores so that the scale had scores with a mean close to 50, a standard deviation close to 10, and items which were uncorrelated. Although both scales contained all 12 items, the physical health measure (SF-12-v2 physical component, range 17.15–77.63) emphasized physical functioning, role functioning, body pain, and general health status over the previous 30 days, while the psychological health measure (SF-12-v2 mental component, range 17.04–77.19) emphasized vitality, social functioning, emotional functioning, and mental health status over the previous 30 days [49].

#### Social support

Social support was measured using the SSRS, which has been shown to have high reliability and validity on a wide range of Chinese populations [51]. The 2 month test–retest reliability of the SSRS has been shown to exceed 0.92 [52].

Social support has been defined as the ‘assistance and protection given to others, especially to individuals’ [53]. The SSRS contains 10 items and measures 3 types of social support; subjective support (SS, 4 items), objective support (OS, 3 items), and support availability (SA, 3 items). Subjective support reflects the perceived interpersonal network that a person can count on, objective support reflects the degree of actual support the person received in the past, and support availability refers to a person’s behavior pattern when seeking social support.

In our study, 10 questions were designed according to Xiao’s 1999 work and were adjusted to the Wenchuan earthquake situation [52]. As with the mental health assessment, higher scores indicated stronger social support. The Cronbach alpha coefficient for social support was 0.91. The subjective support score ranged from 8 to 32 (4 questions), the objective support score ranged from 1 to 22 (3 questions), and the support availability score ranged from 3 to 12 (3 questions). These SSRS item scores were simply added up, with the total support score ranging from 12 to 66 [54].

### Statistical analysis

In this study, data were expressed as a frequency for the nominal variables, and as a mean ± standard deviation (SD) for the continuous variables. Descriptive statistics were used to analyze the demographic variable characteristics of gender, age, ethnicity, income, education level, exposure degree, and housing status. Exposure degree was categorized by the number of the events participants had encountered as a result of the earthquake, and was coded as: low exposure (0–1 event), moderate exposure (2–3 events), and high exposure (more than 4 events). Then, seven risk perception items (risk of earthquake recurrence, risk of secondary disaster occurrence, risk of breakdown in post-seismic physical and mental health, risk of environment, risk of falling into poverty, risk of national culture loss, and risk of unreasonable post-seismic compensation) were applied to a factor analysis, and some abstract factors were extracted based on the explained variance. At the same time, difference tests for ‘Age’ and risk perception were computed, and difference tests between the other demographic variables and risk perception were determined using variance analyses. Demographic characteristics were the control variables, and multiple regression analyses were used to examine the mediating effects of risk perception, the abstract risk factors on the relationship between exposure and psychological health, and the moderating effects of social support on the influence of risk perception on psychological health. For missing data, list wise deletion was used. All tests were 2-tailed, and significance was set at 0.05. All statistical procedures were completed using SPSS16.0 (SPSS, Chicago, IL, USA).

## Results

### Demographic characteristics and exposure

The intention was to interview 2300 households one-year after the earthquake. However, 220 households in the sample areas were not interviewed as some families were unable to remain in their original houses or had been subsequently relocated (*n* = 139; 6.04 %), some refused to participate in the survey (*n* = 38; 1.65 %), some had difficulties understanding Mandarin (*n* = 27; 1.17 %), and some household inhabitants were ineligible (*n* = 16; 0.70 %) as they were mentally challenged or had some other major psychoses such as schizophrenia, depressive disorders or other mental disorders. As a result, the results provided here were based on the data from the remaining 2,080 subjects.

The demographic characteristics and exposure degree in the study sample are shown in Table 1. The majority (59 %) of the subjects were male and at the time of the interview, the mean age was 38.24 ± 8.82 years (ranging from 18 to 68 years). Besides the Han ethnic group (80.5 %), the Tibetan (7.1 %), Qiang (10.1 %), Hui (1.8 %), and other (Tujia and Yi, 0.5 %) ethnic groups participated. For personal income, 83.9 % earned less than 2000 RMB per month. From a door-to-door census conducted 1 year after the earthquake, 30.9 % still lived in prefabricated houses, and 36.4 % continued to live in their original homes. However, a small portion of the sample lived in a rented house or a dormitory. Overall, 53 % of respondents had a relatively low education level.

**Table 1:** Study sample characteristics (*N* = 2,080)

	Number	%
Gender		
Male	1227	59.0
Female	853	41.0
Age		
18–30	441	21.2
31–40	849	40.8
41–50	609	29.3
51–68	181	8.7
Education		
Graduate	47	2.3
Bachelor	938	45.1
No degree	1095	52.6
Exposure degree		
High	392	18.9
Moderate	766	36.8
Low	922	44.3
Ethnic		
Han	1674	80.5
Tibetan	147	7.1
Qiang	211	10.1
Hui	37	1.8
Other	11	0.5
Income		
<1,000	385	18.5
1,000-2,000	1360	65.4
2,000-3,000	264	12.7
>3,000	71	3.4
Housing		
Original house	758	36.4
Public dormitory	295	14.2
Rented house	643	30.9
Temporary settlement	384	18.5

Exposure degrees were categorized by the number of events participants had encountered as a result of the earthquake, and were coded as; low exposure (0–1 event), moderate exposure (2–3 events), and high exposure (more than 4 events). 392 participants (18.9 %) had high exposure, 766 subjects (36.8 %) had moderate exposure and the others (922, 44.3 %) had low exposure.

Physical health, mental health and overall health status were grouped by demographic variables and level of earthquake exposure, from which it was found that males reported higher mental health status scores. Survivors who belonged to the Hui ethnic group, who were between 51–68 years old, had higher education levels, earned more than 3,000 RMB per month, and lived in their original house had the highest scores for all three aspects and were in the low exposure group.

### Risk perception characteristics

Because the individual risk perceptions for different risk items had similar features, we decided to classify earthquake risk items with similar cognitive characteristics as it was easier to analyze survivor risk perception and its role in mental health.

From the survey data, a factor analysis was conducted based on the risk perception degree of the seven items (KMO: 0.88 > 0.8; Bartlett test: *p* = 0.01 < 0.05. The data can be used for factor analysis). Using variance maximization rotation principal component analysis, 2 factors were extracted based on an explained variance of 59. 1 %, the results for which are shown in Table 2. Factor 1 included 4 items: the risk of earthquake recurrence, the risk of secondary disaster occurrence, the risk of post-seismic physical and mental health and the risk of ecological environmental deterioration. Factor 2 included 3 items: the risk of unreasonable post-seismic compensation, the risk of falling into poverty and the risk of losing national culture. Factor 1 related to the direct hurt felt from the earthquake and focused on the visible, physical threats, so could be called “direct risk perception (DRP)”. Factor 2 was mainly related to the invisible, long-term and unpredictable post-earthquake consequences, so was called “indirect risk perception (IRP)”.

**Table 2:** Risk factors Factor analysis

Items	Loading
Direct risks	
Risk of earthquake recurrence	0.801
Risk of secondary disaster occurrence	0.752
Risk of breakdown in post-seismic physical and mental health	0.734
Risk of environment	0.702
Indirect risks	
Risk of falling into poverty	0.822
Risk of national culture loss	0.819
Risk of unreasonable post-seismic compensation	0.807

For the four risk dimensions, direct risk perception was based on the degree of disaster controllability and the seriousness of the consequences, while indirect risk perception was based on the likelihood of occurrence and familiarity.

Grouped by demographic variables and the variables used to assess the level of exposure to the earthquake, the direct and indirect risk scores are shown in Table 3. Females tended to perceive greater direct risk, while males felt more indirect risk. Age was found to have no significant effect on the risk perception level. However, those who resided in their original houses had higher direct risk perception, and those of Han nationality, those with higher education levels and those who earned more than 3000 RMB per month had lower indirect risk scores. For the different levels of exposure, there were significant differences found in both direct risk perception and indirect risk perception, with a higher level of exposure corresponding to a higher risk perception level.

**Table 3:** Scores for direct risk and indirect risk for each socio-demographic variable (*N* = 2,080)

Variables	Direct risk		Indirect risk	
	Mean (SD)	*p* value	Mean (SD)	*p* value
Gender		*		*
Male	52.25 (8.67)		46.74 (8.43)	
Female	54.11 (9.54)		45.03 (8.11)	
Age				
18–30	53.01 (7.88)		44.76 (7.32)	
31–40	52.78 (9.34)		45.23 (7.93)	
41–50	53.34 (8.75)		44.85 (6.22)	
51–68	52.84 (9.99)		45.13 (8.21)	
Income				**
<1,000	53.89 (9.37)		46.28 (6.39)	
1,000–2,000	53.63 (9.82)		45.91 (7.99)	
2,000–3,000	54.18 (9.62)		45.92 (7.36)	
>3,000	53.98 (9.37)		44.25 (6.81)	
Ethnic				**
Han	53.10 (9.42)		43.95 (6.44)	
Tibetan	53.83 (8.88)		45.82 (7.26)	
Qiang	54.12 (9.21)		45.12 (7.29)	
Hui	53.73 (9.56)		45.62 (6.92)	
Other	53.52 (8.59)		46.03 (8.31)	
Education				**
Graduate	53.64 (8.22)		43.63 (6.30)	
Bachelor	54.16 (9.04)		43.89 (6.98)	
No degree	54.42 (8.98)		45.57 (8.28)	
Housing		*		
Original house	53.89 (8.34)		45.23 (7.24)	
Public dormitory	52.43 (7.84)		45.81 (6.32)	
Exposure		***		***
High	55.31 (9.97)		46.75 (8.10)	
Moderate	54.86 (9.74)		45.66 (8.41)	
Low	53.15 (9.43)		44.45 (8.22)	

* < 0.05; ** < 0.01; *** < 0.001

The difference tests for ‘Age’ were computed using a *t* test, while the difference tests between the other subgroups were determined using the analysis of variance (ANOVA)

### Mediating effect of risk perception

The correlation coefficients between level of exposure, risk perception and psychological health are presented in the Table 4. Risk perception was divided into direct risk perception (DRP) and indirect risk perception (IRP). Psychological health was divided into a physical component summary (PCS) and a mental component summary (MCS). Level of exposure and the two risk perception branches were significantly negatively related to the two psychological health domains, as was expected. The DRP was found to be more closely associated with PCS (*r* = −0.21), while the IRP had a stronger correlation with the MCS (*r* = −0.24). The correlation between the level of exposure and the two risk perception dimensions was measured with the highest correlation coefficient (*r* = 0.23) being between level of exposure and the DRP. These results laid the foundation for subsequent hierarchical regression analyses.

**Table 4:** Correlation coefficients between the study variables

	Exposure	RP	DRP	IRP	PH	PCS	MCS
Exposure	1						
RP	0.22**	1					
DRP	0.23**	0.37**	1				
IRP	0.20**	0.35**	0.32**	1			
PH	−0.10**	−0.11**	−0.08*	−0.09*	1		
PCS	−0.12**	−0.09**	−0.21**	−0.04	0.42**	1	
MCS	−0.07**	−0.10**	−0.06*	−0.24**	0.38**	0.56**	1

* < 0.05; ** < 0.01

RP Risk perception, PH psychological health

For an examination of the mediating effect of risk perception on earthquake-related exposure and psychological health, first, risk perception and psychological health were the dependent variables and level of exposure was the independent variable, then, psychological health was the dependent variable and level of exposure and risk perception were the independent variables. Controlling for demographic variables (gender, age, ethnicity, housing status, income level and education level), the results of the regression analyses are presented in Table 5.

**Table 5:** Results of the hierarchical regression analysis: validation of the mediating effect of risk perception

Variables	Step1				Step2	
	PH		RP		PH	
Control variables						
Gender	−0.206*	−0.121	−0.094	−0.106	−0.206*	−0.157*
Age	0.123	0.098	0.000	−0.014	0.123	0.102
Ethnic	−0.054	−0.071	0.051	0.019	−0.054	−0.065
Education	0.178	0.098	−0.093	−0.105	0.178	0.121
Income	0.165	0.127	−0.097	−0.084	0.165	0.148
Housing	−0.154	−0.158*	0.052	0.036	−0.154	−0.140
Independent variables						
Exposure		−0.167***		0.298***		−0.132**
Mediating variables						
RP						−0.331***
F	1.764	95.671***	4.553***	91.352***	1.413	122.910***
R^2^	0.005	0.135	0.049	0.161	0.005	0.301
△R^2^	0.012	0.129***	0.057***	0.123***	0.015	0.164***

* < 0.05; ** < 0.01; *** < 0.001

B unstandardized, beta standardized coefficients, R^2^ explanation rate, △R^2^ change in explanation rate in each step

The first step examined the effect of exposure on psychological health. Table 5 shows that after controlling for the demographic variables, level of exposure was found to significantly affect psychological health, with the standardized regression coefficient being −0.167 (*p* < 0.001).

At the same time, the influence of the level of exposure on risk perception was tested. The results showed a significant effect between exposure and risk perception, with the standardized regression coefficients being 0.298 (*p* < 0.001).

The second step incorporated the level of exposure and risk perception into the regression equation to explain psychological health. From this it was found that the effect of risk perception was significant, with the standardized regression coefficient being −0.331 (*p* < 0.001). However, the effect of the level of exposure was still significant, with the standardized regression coefficient being −0.132 (*p* < 0.01). However, in comparison with the results in the first step, the prediction effect of the level of exposure to psychological health was weakened, so it was concluded that risk perception had a partial mediating effect on the relationship between exposure and psychological health.

To further understand the intermediary roles of the DRP and IRP on the relationship between exposure and the two psychological health domains (i.e. PCS and MCS), we first controlled the demographic variables, then examined the influence of exposure on PCS (−0.201, *p* < 0.001), DRP (−0.369, *p* < 0.001), with the results showing that these were significant influences (Table 6). The exposure and DRP were then incorporated into the regression equation to explain the PCS, with the results showing that the DRP effect was significant and the standardized regression coefficient was 0.402 (*p* < 0.001). The effect of exposure was also significant, with the standardized regression coefficient being 0.161 (*p* < 0.001), but compared to the previous step, the prediction effect of exposure to PCS was weakened, so can be concluded that the DRP had a partial mediating effect on the relationship between exposure and PCS.

**Table 6:** Results of hierarchical regression analysis: validation of mediating effect of DRP on PCS

Variables	Step1				Step2	
	PCS		DRP		PCS	
Control variables						
Gender	0.158*	0.122	−0.175**	−0.166**	0.158*	0.163*
Age	0.037	0.045	0.008	0.000	0.037	0.033
Ethnic group	0.000	0.011	0.021	0.049	0.000	0.000
Education	0.068	0.047	0.037	0.034	0.068	0.102
Income	−0.106	−0.126	0.095	0.103	−0.106	−0.115
Housing	−0.013	−0.054	−0.143	−0.126	−0.013	−0.025
Independent variables						
Exposure		−0.201***		0.369***		−0.161***
Mediating variables						
DRP						−0.402***
F	2.213	101.457***	6.352***	90.385***	2.213	91.352***
R^2^	0.012	0.190	0.049	0.201	0.012	0.323
△R^2^	0.028	0.154***	0.093***	0.186***	0.028	0.180***

* < 0.05; ** < 0.01; *** < 0.001

B unstandardized, beta standardized coefficients, R^2^ explanation rate, △R^2^ change in explanation rate in each step

The same steps were used in the examination of the mediating effect of IRP between exposure and PCS. However, no significant effect was found.

Using the same steps, we also examined the mediating roles of DRP and IRP in the relationship between level of exposure and the MCS, with the results showing a partial intermediary role for both the DRP and the IRP.

### Moderating effect of social support

The correlation coefficients between risk perception, DRP, IRP, psychological health, PCS, MCS and social support are presented in Table 7. Three types of social support; were estimated; subjective support (SS), objective support (OS) and support availability (SA). The results showed that the correlation coefficients between these variables reached a significant level (*p* < 0.01). The highest correlation coefficient (*r* = 0.47) was found between subjective support and support availability. These results were then used in the next hierarchical regression analysis using the above variables.

**Table 7:** Correlation coefficients between the study variables

	DRP	IRP	SS	OS	SA	PCS	MCS
DRP	1						
IRP	0.32**	1					
SS	0.06**	0.22**	1				
OS	0.03**	0.09**	0.27**	1			
SA	0.11**	0.12**	0.47**	0.38**	1		
PCS	0.23**	0.07**	0.24**	0.17**	0.16**	1	
MCS	0.12**	0.16**	0.08**	0.04**	0.27**	0.56**	1

* < 0.05; ** < 0.01

For an examination of the moderating effect of social support on risk perception and psychological health, first we tested for the interaction effect between risk perception and social support. Then, using psychological health as the dependent variable, the demographic variables as the control variables, and risk perception, social support and the interaction effect between risk perception and social support as the independent variables, we conducted a hierarchical regression analysis, the results of which are in Table 8.

**Table 8:** Interaction effect analysis for the total PH score (*N* = 2,080)

Variables	PH				
	B	SE	Beta	△R^2^	R^2^
Step 1				0.063**	0.063**
Gender	−0.808	0.164	−0.136**		
Age	0.134	0.098	0.041		
Ethnic group	−0.059	0.121	0.013		
Education	0.479	0.152	0.090**		
Income	0.666	0.130	0.154**		
Housing	−0.156	0.067	0.064*		
Step 2				0.159**	0.223**
RP	−0.128	0.014	−0.322**		
Step 3				0.020**	0.243**
SS	0.107	0.009	0.154**		
Step 4				0.011**	0.254**
RP × SS	−0.022	0.002	−0.085**		

* < 0.05; ** < 0.01

B unstandardized, beta standardized coefficients, R^2^ explanation rate, △R^2^ change in explanation rate in each step

RP Risk perception, SS social support, PH psychological health

Survivors who were male, had a high level of education, a high income and better housing were found to have a higher level of psychological health. These demographic characteristics explained a 6.3 % change in the psychological health (△R^2^ = 0.063, *p* < 0.01). In Step 2, risk perception explained a 15.9 % change in psychological health (△R^2^ = 0.159, *p* < 0.01); namely a lower psychological health score could be explained by a higher level of risk perception. In Step 3, we found that the level of social support also positively contributed to the explanation (△R^2^ = 0.020, *p* < 0.01). In the last step, we examined the interaction effect, from which it was found risk perception and social support significantly contributed to the explained variance (△R^2^ = 0.011, *p* < 0.01). Therefore, our hypotheses were supported by the results. It was proven that risk perception had a negative effect on psychological health and that social support was a moderator for the negative effects of risk perception on psychological health.

Using similar steps, with PCS and MCS as the dependent variables, the results of the regression analyses are presented in Tables 9 and 10.

**Table 9:** Interaction effect analysis for total the PCS score (*N* = 2,080)

Variables	PCS				
	B	SE	Beta	△R^2^	R^2^
Step 1				0.048**	0.048**
Gender	0.820	0.112	0.174		
Age	0.100	0.066	0.038		
Ethnic group	0.031	0.083	0.009		
Education	0.257	0.013	0.061		
Income	0.605	0.103	0.176**		
Housing	−0.062	0.044	−0.032*		
Step 2				0.135**	0.184**
DRP	−0.129	0.018	−0.360**		
IRP	−0.042	0.002	−0.033		
Step 3				0.046**	0.230**
SS	0.173	0.021	0.184**		
OS	0.049	0.002	0.034		
SA	0.102	0.012	0.058		
Step 4				0.008**	0.238**
DRP × SS	0.044	0.010	0.045**		
DRP × OS	0.005	0.001	0.024		
DRP × SA	0.013	0.004	0.014		
IRP × SS	0.004	0.003	0.023		
IRP × OS	0.029	0.003	0.004		
IRP × SA	0.007	0.011	0.024		

* < 0.05; ** < 0.01

B unstandardized, beta standardized coefficients, R^2^ explanation rate, △R^2^ change in explanation rate in each step

**Table 10:** Interaction effect analysis for the total MCS score (*N* = 2,080)

Variables	MCS				
	B	SE	Beta	△R^2^	R^2^
Step 1				0.068**	0.068**
Gender	−0.806	0.163	−0.135**		
Age	0.129	0.096	0.039		
Ethnic group	−0.075	0.121	−0.017		
Education	0.468	0.151	0.088		
Income	0.951	0.150	0.220**		
Housing	−0.151	0.067	−0.062*		
Step 2				0.149**	0.218**
DRP	−0.106	0.010	−0.123**		
IRP	−0.134	0.011	−0.137**		
Step 3				0.038**	0.256**
SS	0.113	0.002	0.075**		
OS	0.120	0.014	0.130**		
SA	0.098	0.009	0.064**		
Step 4				0.016**	0.273**
DRP × SS	0.053	0.004	0.052**		
DRP × OS	0.024	0.002	0.013		
DRP × SA	0.015	0.010	0.045		
IRP × SS	0.010	0.009	0.026		
IRP × OS	0.042	0.003	0.046**		
IRP × SA	0.063	0.012	0.054**		

* < 0.05; ** < 0.01

B unstandardized, beta standardized coefficients, R^2^ explanation rate, △R^2^ change in explanation rate in each step

Survivor risk perception was found to make a significant contribution to PCS, explaining 13.5 % of the change (△R^2^ = 0.135, *p* < 0.01). For the two risk perception dimensions, lower PCS scores were explained by a high level of DRP rather than IRP. The next step showed that subjective support had a positive effect on PCS (△R^2^ = 0.046, *p* < 0.01). For the interaction effect, a lower degree of DRP with a higher level of subjective support was found to be related to a higher level of PCS (△R^2^ = 0.008, *p* < 0.01). Survivors who had received greater subjective support were found to be protected from the negative effects of DRP on PCS.

A lower MCS score was found to be related to a higher level of risk perception (△R^2^ = 0.149, *p* < 0.01), and a higher degree of both DRP and IRP were found to contribute to a lower MCS score. At the same time, a better MCS was explained by a higher level of subjective support, objective support and support availability (△R^2^ = 0.038, *p* < 0.01). For the interaction effect, a higher DRP degree with a lower subjective support predicated a poorer MCS, while a lower level of IRP with a higher level of objective support was related to a higher MCS level. In addition, the interaction between IRP and support availability made a significant contribution to the explained variance in MCS (△R^2^ = 0.016, *p* < 0.01). Survivors who received more subjective support were protected from the negative effects of DRP on MCS, and those who received more objective support and support availability were protected from the negative effects of IRP on MCS.

## Discussion

This study investigated survivor risk perception and its mediating effect between exposure level and psychological health status, and also examined the moderating role social support had on the relationship between risk perception and psychological health. The results indicated that earthquake-exposure directly impacted survivors’ psychological health, and risk perception had an indirect impact. However, survivors' risk perception only had a partial mediating effect between exposure and psychological health. More specifically, exposure was partially affected by direct risk perception (DRP), which affected the two psychological health domains (PCS and MCS), and partially affected by indirect risk perception (IRP), which influenced MCS. Social support has been demonstrated to mitigate the influence of risk perception on psychological health. In this paper, subjective support was found to moderate the effect of DRP on PCS and the effect of IRP on MCS. Objective support and support availability moderated the effect of IRP on MCS.

Many studies have documented post-disaster psychological health risk factors, which has been associated with a range of factors including socio-demographic and background factors, event exposure characteristics, social support factors and personality traits [9, 27]. Many associated risk factors have been identified in cross-sectional studies: high exposure [55, 56], being female [57, 58], being middle-aged [25], having a low income [59], having a low educational level [60], and risk perception [12]. Many of our findings are consistent with previous studies, while some varied because of the earthquake intensity, the types of exposure, the sampling selection, the time elapsed since the earthquake, and the measures used. Some studies have reported that older people are vulnerable in disaster events [25, 43, 44]; however, this study found that older people (aged 51–68) had a high psychological health score, while the middle-aged (aged 35–44) were found to have a higher risk of poor psychological health. One possible explanation for this result is the burden perspective hypothesis which suggests that middle-aged adults have a greater burden than others [23, 60]. This article is different from previous studies also because the risk perceptions were taken as intervening variables for the relationship between earthquake exposure and psychological health. Although studies on the risk perception dichotomy (dreadfulness and the unknown) are common, few previous studies have divided these risk items into direct and indirect factors, which is closer to the actual situation. With this more specific knowledge, different risk communication strategies can be designed.

A questionnaire related to social, economic and ecological factors was used to survey survivors one year after the Wenchuan earthquake. Several risk items and four attributes were included; disaster controllability, seriousness of the consequences, occurrence likelihood and familiarity. Questionnaire reliability and validity was checked in a pilot test, from which two additional abstract properties were identified; direct risk perception, which included the risk of earthquake recurrence, secondary disaster occurrences, post-seismic physical and mental health and the environment; and indirect risk perception, which was made up of the risk of falling into poverty, national culture loss and unreasonable post-seismic compensation. These two abstract terms emphasized different attributes, with the DRP highlighting the dread of risk events, and the IRP highlighting the unpredictability of future risk events.

For survivor risk perception, a higher degree of exposure meant higher levels of both DRP and IRP. Ho et al. found that previous disaster exposure was a good predictor of a survivor’s attitude toward natural hazards [14]. The earthquake had a significant impact, and one year after the event, these fears had not been completely eliminated. The perceived risk of emergency events like earthquakes has been found to be heightened when the event had been previously experienced [36] as the survivors’ knowledge and awareness is more specific and profound because of the earthquake experience. Survivors who have had higher exposure to the event also often suffer a greater loss of property, possessions and social networks. Therefore they are more likely to feel greater uncertainty about their future. From the demographic variables, the results showed that females had greater DRP, and males had greater IRP. A possible explanation for these results was the difference in gender for the subjective seismic exposure measures [23] as the females might have experienced greater levels of fear and anxiety regarding their own safety and that of their family members so perceived greater DRP. However, males probably perceived greater IRP because of their societal (e.g. working) and family (e.g. often providing support to both children and parents) responsibilities. Armas found that earthquake risk perception differed with gender, age, education, residential area and socioeconomic status, seismic characteristics and degree of exposure, with the most vulnerable found to be the older aged population [21]. In this study, however, no significant differences in risk perception levels were found for age, which may be because no matter their age, people feel the same level of dread or fear about the unknown future. Survivors of Han nationality and those who had a high education level and a high monthly income showed a lower level of indirect risk. The Han nationality was the most populous ethnic group in the affected area, with most having better living conditions. Survivors who had higher levels of education and higher monthly incomes also probably had better life skills than others before the event so were not excessively worried about their future survival. Interestingly, however, the interviewed survivors who had been living in their original housing were found to have a higher DRP. A possible explanation for this is that they may have seen increased death and injury from the collapse of the buildings of others and their houses may also have been damaged in the event, so that they may have had greater worries about their safety.

This study examined the mediating effect of survivors' risk perception on earthquake related exposure and psychological health, and the results showed that while exposure directly led to psychological health problems, these were also by affected the survivors’ risk perception. More specifically, the DRP was found to have a mediating role between exposure for both PCS and MCS, while the IRP only had a mediating effect on exposure and MCS. A possible explanation is that greater exposure imbued in the survivors a greater feeling of dread about a future recurrence or secondary disaster, which increased their DRP. Therefore, if these people remained in this high-risk environment for a long time, they could easily suffer from serious stress, anxiety, insomnia [61] or other symptoms, further decreasing their physical health levels. When taking the other variables into the regression analysis and with the MCS as a dependent variable, the results showed that both the DRP and IRP had mediating effects on the relationship between exposure and MCS. This was found to be because the level of exposure influenced survivors’ psychological health as well as the two mediators, the DRP and IRP. Earthquake experiences lead to fear about the deaths and injuries which resulted from the event, so survivors connect the earthquake with death, panic, fear and nervousness. Because this shock caused serious damage and significant changes, there was a huge uncertainty about the future [62], and in cases where there were serious consequences, this uncertainty put people in a state of depression and anxiety [35], thereby causing enormous psychological pressure. Therefore, reducing risk perception is the key to restoring survivors’ mental health. However, our study found that risk perception was found to play only a partial intermediary role in the relationship between exposure and psychological health; therefore, except for risk perception, exposure was found to be more likely to be affected by other variables that influence psychological health, an area that is worthy of further study.

To examine the moderating effect of social support on the relation between risk perception and psychological health, this research also used a hierarchical regression analysis on the data. The results showed that the interactive effect of risk perception and social support reached significant levels in the regression equation, which proved that there was a moderating effect on the relationship. Further, we verified that subjective support had a moderating effect on the effect of DRP on both PCS and MCS, while objective support and support availability were found to be moderators between IRP and MCS. A possible explanation for this is that the DRP is mainly related to the uncontrollability of earthquake shock on survivors and the seriousness of the consequences. Most survivors tend to feel helpless and overwhelmed in the face of natural disasters, and people exposed to such a severe disaster are equal no matter their material base or social standing. However, subjective support was found to be a mediating variable influencing both psychology and behavior [63] and affecting some coping strategies, such as the resistance, avoidance and fantasy often used to avoid the fear that can lead to further physical and psychological pressure, and somewhat mitigating the psychological reaction caused by worry.

Unlike the DRP, the IRP focused more on aspects related to the post-disaster situations, such as the economic and ecological environments, reconstruction and culture, so survivors with different levels of objective support are not equal, as objective support is based on the complexity of social background, social networks and resource conditions. However, the IRP is also related to a future which has no clear direction. However, objective support to some extent reflected the survivors’ position in the social network and the potential of social resources, which are the necessary resources for post-earthquake economic, social and ecological recovery. People who have more extensive social relations may be able to obtain and a greater amount of accurate information faster than others, and thus may be more likely to recover more quickly. Greater support availability has been found to increase the enthusiasm of survivors participating in various social activities [64]. Survivors with high support availability receive more social, economic, and ecological support.

This study provides information about two ways to alleviate the psychological health problems in earthquake survivors. The first is to prevent the effect of exposure on psychological health by reducing risk perception levels, which was found to be an important mediating variable. The second is to mitigate the influence of risk perception on psychological health through increased social support. To reduce risk perception, adequate communication about the potential risks should be given. In this article, the risk perception of the earthquake survivors was divided into direct and indirect risk perception, which respectively reflected the dreadfulness of casualties, and the threats to personal safety resulting from the earthquake, and the unknown future in terms of the economic, societal and ecological environments.

The uncontrollability of events and the severity of the consequences are prominent features of dread, so risk communication needs to be viewed along two different paths. The first is to reduce the perceived uncontrollability of the earthquake shock to the survivors and the second is to decrease the perceived severity, especially for females. Earthquake education could be strengthened to inform people that the damage can be reduced using effective measures to make people feel more secure, such as scientific escape and suspension measures and the reinforcement of housing facilities. In addition, information about casualties, aftershocks and secondary disasters reflect the severity of the consequences caused by the earthquake and have a major impact on survivors’ risk perception. Although the number of casualties cannot be changed, governments and related departments could reduce the number of casualties using high-efficiency relief measures. Because aftershocks frequently occur, the government and relevant departments need to guide the public to safer places or provide prefabricated houses and other means to reduce the possible damage to reduce the DRP levels.

For most survivors, this was the first time they had experienced such a catastrophe, so they lacked any comparative experiences. Faced with post-disaster social, economic, and ecological environmental changes, they were unable to predict what would happen in the future; but poverty, compensation distribution, ethnic culture and ecological environmental damage were some the imagined problems. Therefore, developing effective economic and employment policies, developing ethnic cultural protection schemes, providing transparent relief support policies and adequate insurance compensation, as well as ecological environmental protection is a key priority. Strengthening scientific research investment to improve the level of earthquake prediction, and disseminating earthquake prevention knowledge to the general public could enhance the public's sense of controllability, thereby reducing the IRP level.

In addition to risk management to improve the mental health of survivors, the fact that social support moderates the risk perception influence on psychological health also indicates the need for clinical measures for survivors with mental health symptoms. The results showed a detailed mechanism for the moderating effects of different dimensions of social support and the complex relations between them. Therefore, it is important to develop appropriate social support strategies for mental health improvements in survivors, and provide longer term social support as part of long-term mental health care policies for earthquake survivors. In all, the social support provided by the government and the society to survivors with PTSD should not only focus on objective support, but should consider a more comprehensive approach. Helping survivors establish and maintain supportive social relationships through the provision of more support networks and more ways to socially participate would enhance self-confidence and personal skills and encourage increased subjective support. Finally, mental care policies should also focus on survivors who are female, have a lower level of education, have a lower level of income, live in worse housing and have a higher level of exposure to the earthquake.

## Conclusion

In a word, taking the 2008 Wenchuan earthquake as an example, we attempted to apply the results of our study to possible mental health interventions in similar future disasters. Research has shown that in the emergency management of disasters such as earthquakes, it is necessary have adequate risk communication. Through the implementation of reasonable regulations and by controlling risk perception levels and its influencing factors, an emergency risk communication management system could be established to encourage rational risk consciousness to assist people avoid or mitigate the impact of disasters on their psychological health, restore them to a healthy psychological state and restore confidence in their life and the future.

Several limitations to this study should be noted. First, because of a lack of data from before the earthquake, it cannot be assumed that all the risk perception levels reported were a direct result of the earthquake. Second, a survey in non-disaster areas was not conducted, thus a comparative study based on regional differences was not available. Another limitation of the study was that the variance explained by the social support remained rather low in part of the regression models. This suggested that it would be useful to broaden the domain of examined variables, so other psychological impairments could be taken into account in future studies or other moderator factors could be incorporated into the models. Fourth, selection bias may exist in the data because of the sampling and recruitment method. Last, a self-report instrument was used, so the participants may have over or under reported.

## Availability of data and materials

The datasets supporting the conclusions in this article are included within the article.

## Abbreviations

PTSD: post-traumatic stress disorder
SF-12-v2: short Form-12, version 2
SSRS: social support rating scale
DSM-IV: diagnostic and statistical manual of mental disorders, fourth edition
PCS: physical component summary
MCS: mental component summary
SS: subjective support
OS: objective support
SA: support availability
SD: Standard Deviation
DRP: direct risk perception
IRP: indirect risk perception

## References

[1] Collins PY, Patel V, Joestl SS (2011). Grand challenges in global mental health[J]. Nature.

[2] Schnurr P P, Green B L. Trauma and health: Physical health consequences of exposure to extreme stress[J]. Journal of Health Psychology. 2004;10(9):734–736.

[3] Ben-Ezra M. Traumatic events take their toll on mental health[J]. Nature. 2004;430(7000):611–1. 10.1038/430611a 15295570

[4] McMillen JC, Smith EM, Fisher RH (1997). Perceived benefit and mental health after three types of disaster[J]. J Consult Clin Psychol.

[5] Chou FHC, Chou P, Su TTP (2004). Quality of life and related risk factors in a Taiwanese Village population 21 months after an earthquake[J]. Aust N Z J Psychiatry.

[6] Xu J, He Y (2012). Psychological health and coping strategy among survivors in the year following the 2008 Wenchuan earthquake[J]. Psychiatry Clin Neurosci.

[7] Rhoads J, Pearman T, Rick S (2007). Clinical presentation and therapeutic interventions for posttraumatic stress disorder post-Katrina[J]. Arch Psychiatr Nurs.

[8] Rateau MR (2009). Differences in emotional well-being of hurricane survivors: A secondary analysis of the ABC news Hurricane Katrina anniversary poll[J]. Arch Psychiatr Nurs.

[9] Brewin CR, Andrews B, Valentine JD (2000). Meta-analysis of risk factors for posttraumatic stress disorder in trauma-exposed adults. J Consult Clin Psychol.

[10] Carson MA, Paulus LA, Lasko NB, Metzger LJ, Wolfe J, Orr SP, Pitman RK (2000). Psychophysiologic assessment of posttraumatic stress disorder in Vietnam nurse veterans who witnessed injury or death. J Consult Clin Psychol.

[11] Xu J, Song X (2011). Posttraumatic stress disorder among survivors of the Wenchuan earthquake 1 year after: prevalence and risk factors[J]. Compr Psychiatry.

[12] Wu P, Fang Y, Guan Z (2009). The psychological impact of the SARS epidemic on hospital employees in China: exposure, risk perception, and altruistic acceptance of risk[J]. Can J Psychiatry.

[13] La Greca AM, Silverman WK, Lai B (2010). Hurricane-related exposure experiences and stressors, other life events, and social support: Concurrent and prospective impact on children's persistent posttraumatic stress symptoms[J]. J Consult Clin Psychol.

[14] Ho MC, Shaw D, Lin S (2008). How do disaster characteristics influence risk perception?[J]. Risk Anal.

[15] Lowe SR, Chan CS, Rhodes JE (2010). Pre-hurricane perceived social support protects against psychological distress: a longitudinal analysis of low-income mothers[J]. J Consult Clin Psychol.

[16] Kobbeltved T, Brun W, Johnsen BH (2005). Risk as feelings or risk and feelings? A cross-lagged panel analysis[J]. J Risk Res.

[17] Tseng MCM, Lin YP, Hu FC (2013). Risks perception of electromagnetic fields in Taiwan: The influence of psychopathology and the degree of sensitivity to electromagnetic fields[J]. Risk Anal.

[18] McArdle SC, Rosoff H, John RS (2012). The Dynamics of evolving beliefs, concerns emotions, and behavioral avoidance following 9/11: a longitudinal analysis of representative archival samples[J]. Risk Anal.

[19] Slovic P (1987). Perception of risk[J]. Science.

[20] Slovic P (1999). Trust, emotion, sex, politics, and science: Surveying the risk-assessment battlefield[J]. Risk Anal.

[21] Armaş I (2006). Earthquake risk perception in Bucharest, Romania[J]. Risk Anal.

[22] Gierlach E, Belsher BE, Beutler LE (2010). Cross‐cultural differences in risk perceptions of disasters[J]. Risk Anal.

[23] Karanci AN, RÜSTEMLI A (1995). Psychological consequences of the 1992 Erzincan (Turkey) earthquake[J]. Disasters.

[24] Basoglu M, Salcioglu E, Livanou M (2002). Traumatic stress responses in earthquake survivors in Turkey[J]. J Trauma Stress.

[25] Thompson MP, Norris FH, Hanacek B (1993). Age differences in the psychological consequences of Hurricane Hugo[J]. Psychol Aging.

[26] Salcioglu E, Basoglu M, Livanou M (2007). Post‐traumatic stress disorder and comorbid depression among survivors of the 1999 earthquake in Turkey[J]. Disasters.

[27] Neria Y, Nandi A, Galea S (2008). Post-traumatic stress disorder following disasters: a systematic review[J]. Psychol Med.

[28] Naeem F, Ayub M, Masood K (2011). Prevalence and psychosocial risk factors of PTSD: 18 months after Kashmir earthquake in Pakistan[J]. J Affect Disord.

[29] Feder A, Ahmad S, Lee EJ (2013). Coping and PTSD symptoms in Pakistani earthquake survivors: Purpose in life, religious coping and social support[J]. J Affect Disord.

[30] Slovic P E. The perception of risk[M]. London: Earthscan publications; 2000.

[31] Christman NJ, McConnell EA, Pfeiffer C (1988). Uncertainty, coping, and distress following myocardial infarction: Transition from hospital to home[J]. Res Nurs Health.

[32] Mishel MH (1984). Perceived uncertainty and stress in illness[J]. Res Nurs Health.

[33] Webster KK, Christman NJ, Mishel MH (1988). Perceived uncertainty and coping post myocardial infarction[J]. West J Nurs Res.

[34] Wineman NM (1990). Adaptation to multiple sclerosis: The role of social support, functional disability, and perceived uncertainty[J]. Nurs Res.

[35] Cho J, Lee J (2006). An integrated model of risk and risk-reducing strategies[J]. J Bus Res.

[36] Knuth D, Kehl D, Hulse L (2014). Risk Perception, Experience, and Objective Risk: A Cross‐National Study with European Emergency Survivors[J]. Risk Anal.

[37] Bland SH, O'leary ES, Farinaro E (1997). Social network disturbances and psychological distress following earthquake evacuation[J]. J Nerv Ment Dis.

[38] Wang X, Gao L, Zhang H (2000). Post‐earthquake quality of life and psychological well‐being: Longitudinal evaluation in a rural community sample in northern China[J]. Psychiatry Clin Neurosci.

[39] Altindag A, Ozen S, Sir A (2005). One-year follow-up study of posttraumatic stress disorder among earthquake survivors in Turkey[J]. Compr Psychiatry.

[40] Zhao C, Wu Z, Xu J (2013). The association between post-traumatic stress disorder symptoms and the quality of life among Wenchuan earthquake survivors: the role of social support as a moderator[J]. Qual Life Res.

[41] Maltais D, Lachance L, Brassard A (2005). Social support, coping and psychological health after a flood[J]. Sciences Sociales et Sante.

[42] Zheng R, Shi K, Li S (2009). The Influence factors and mechanism of societal risk perception[M]//Complex sciences.

[43] Ticehurst S, Webster RA, Carr VJ (1996). The psychosocial impact of an earthquake on the elderly[J]. Int J Geriatr Psychiatry.

[44] Phifer JF (1990). Psychological distress and somatic symptoms after natural disaster: differential vulnerability among older adults[J]. Psychol Aging.

[45] Slovic P. Perception of risk: Reflections on the psychometric paradigm[J]. In Social Theories of Risk, S. Krimsky and D. Golding, S. Krimsky and D. Golding^Editors. New York: Praeger. 1992;117-152.

[46] Lam CLK, Eileen YY, Gandek B (2005). Is the standard SF-12 health survey valid and equivalent for a Chinese population?[J]. Qual Life Res.

[47] Fong DYT, Lam CLK, Mak KK (2010). The Short Form-12 Health Survey was a valid instrument in Chinese adolescents[J]. J Clin Epidemiol.

[48] Burdine JN, Felix MR, Abel AL (2000). The SF-12 as a population health measure: an exploratory examination of potential for application[J]. Health Serv Res.

[49] Ware JE, Kosinski M, Turner-Bowker DM (2002). How to score version 2 of the SF-12-v2 health survey Boston[J].

[50] Fleishman JA, Lawrence WF (2003). Demographic variation in SF-12 scores: true differences or differential item functioning?[J]. Med Care.

[51] Xie RH, He G, Koszycki D (2009). Prenatal social support, postnatal social support, and postpartum depression[J]. Ann Epidemiol.

[52] Xiao S (1999). The Social Support Rating Scale[J]. Chin Ment Health J.

[53] Shumaker SA, Brownell A (1984). Toward a theory of social support: Closing conceptual gaps[J]. J Soc Issues.

[54] Ke X, Liu C, Li N (2010). Social support and quality of life: a cross-sectional study on survivors eight months after the 2008 Wenchuan earthquake[J]. BMC Public Health.

[55] Briere J, Elliott D (2000). Prevalence, characteristics, and long-term sequelae of natural disaster exposure in the general population[J]. J Trauma Stress.

[56] North R D. Risk: The Human Adventure[M]. Cambridge: The European Science and Environment Foundation. 2001.

[57] Blain LM, Galovski TE, Robinson T (2010). Gender differences in recovery from posttraumatic stress disorder: A critical review[J]. Aggress Violent Behav.

[58] Matud MP (2004). Gender differences in stress and coping styles[J]. Personal Individ Differ.

[59] Norris FH, Friedman MJ, Watson PJ (2002). 60,000 disaster victims speak: Part I. An empirical review of the empirical literature, 1981—2001[J]. Psychiatry.

[60] Tural Ü, Coşkun B, Önder E (2004). Psychological consequences of the 1999 earthquake in Turkey[J]. J Trauma Stress.

[61] Geng F, Xu T, Wang Y (2013). Developmental trajectories of schizotypal personality disorder-like behavioural manifestations: a two-year longitudinal prospective study of college students[J]. BMC Psychiatry.

[62] Yates JF, Stone ER (1992). Risk appraisal[J]. Risk Taking Behav.

[63] Thoits PA (1983). Multiple identities and psychological well-being: A reformulation and test of the social isolation hypothesis[J]. Am Sociol Rev.

[64] Watanabe C, Okumura J, Chiu TY (2004). Social support and depressive symptoms among displaced older adults following the 1999 Taiwan earthquake[J]. J Trauma Stress.

[65] Babbie ER (1998). The practice of social research[M].

[66] Charan J, Biswas T (2013). How to calculate sample size for different study designs in medical research?[J]. Indian J Psychol Med.

[67] Lench H, Levine L (2005). Effects of fear on risk and control judgements and memory: Implications for health promotion messages[J]. Cognition Emotion.

[68] Öhman A, Mineka S (2001). Fears, phobias, and preparedness: toward an evolved module of fear and fear learning[J]. Psychol Rev.

